# Modern Treatment of Acute Inflammation of the Middle Ear

**Published:** 1896-10

**Authors:** J. A. Mullen

**Affiliations:** 602 Main Street; Houston, Texas


For Texas Medical Journal.

### Modern Treatment of Acute Inflammation of the Middle Ear.

J. A. MULLEN, M. D., HOUSTON, TEXAS.

[Read at the June meeting of the Austin District Medical Society.]

A COMPLETE history of the diagnosis, prognosis and treatment of inflammations of the middle ear would fill volumes and consume too much time that could be used for far more practical purposes; so our object in this paper is merely to present the clinical picture of the common forms of acute tympanic inflammations, and direct attention to their rational and curative treatment. The objects which any form of treatment should have are the complete preservation of hearing, keeping the inflammatory process confined to the middle ear, and the prevention of a chronic purulent discharge, which is the cause of 50 per cent of all cerebral abscesses.

The cases presented will illustrate two very prevalent causes of acute middle ear inflammation, and at the same time demonstrate the curative advance made in the treatment of these conditions within the recollection of those gentlemen present.

Case 1.—Miss G., aged 20 years, Jewess. She had suffered from acute pain in the right ear for five days, with accompanying complete deafness. The history shows that on May 5th the windows of the bed room had been open all night, creating a draught across the bed in which she was sleeping. The next morning she complained of ear-ache, which gradually became more intense with irregular exaccerbations, always worse at night. The membrana tympanum was decidedly inflamed and perceptibly bulging into the external auditory canal, the posterior wall of which was swollen and soggy. Mastoid painful on pressure and slightly œdematous. Temperature 104½°. Bowels constipated. Right sided vertigo.

Treatment.—The drum was incised in its posterior quadrant, permitting a free evacuation of pus. Small doses of calomel and soda bicarb. were ordered every hour. Inflation of middle ear with iodine, camphor and chloroform vapors. Free irrigation with hot sterilized water, instillation of $H_2O_2$, followed by hot water; thoroughly cleansing the tympanic cavity. Dry heat to the auricle and two leeches on the mastoid. Next day temperature 101°; very little ear-pain; free discharge of pus; no mastoid œdema, nor sogging of posterior wall greatly lessened. One week after incision of drum all symptoms had gone, except deafness. Politzer's method of inflation used every day, with the above mentioned vapors, supplemented by massage of the membrana tympanum at each sitting. June 15, hearing for watch 30-40, whispers 7-10.

Case 2.—This case demonstrates the value of early treatment. Miss G. C., aged 10 years, complained on February 9th of acute pain in left ear, which pain had raged all night. Next day, in my office, her temperature was 104°, pulse 128. Bowels constipated; breath foul; drum acutely inflamed; no bulging; only slight mastoid tenderness on deep pressure. She had been sleeping with open windows the night before attacked.

Treatment.—Same as previous case. On the 24th instant hearing for watch 40-40, whispers 9 feet.

Case 3.—Mr. A. M. 25 years of age, had gone bathing on the 11th of September, and dived from a swinging trapeze, a distance of 15 feet, falling on the left side of the head. Blood and serum flowed from left ear immediately after accident. On the fourth day after, when I first saw him, there were pain and a bloody purulent discharge present. The drum was found ruptured just posterior to the handle of the mallelous, and also retracted. The tear was not sufficiently large nor in the right position to permit free drainage. The rent in the drum enlarged to accomplish the latter object. The mastoid was markedly tender and somewhat œdematous.

Treatment.—Same as previous cases, using hot water and $H_2 O_2$ every hour. On the 19th instant temperature 99°, with decrease in severity of all symptoms. On the 20th all symptoms intensified; temperature 103°; mastoid decidedly tender and œdematous; sagging of the posterior wall of the external auditory canal. Leeches applied to mastoid, irrigations of hot boriic acid solution every hour and dry hot applications externally, with the internal administration of Rochelle's salts. The symptoms subsided in twelve hours, thereby doing away with the necesity of a mastoid osteatomy, which had been seriously considered. December 1st, hearing for watch 20-40, whispers heard 5-10.

Case 4.—Mr. D. T., aged 20 years. Three days before consultation, and while in bathing, he experienced a feeling of fullness and pain in the left ear. The pain increased in severity during the night, but less severe during the day. The membrana tympanum showed distinct evidences of inflammation. No mastoid tenderness or œdema. Temperature 101°.

Treatment.—Same as previous cases. Pain and soreness had ceased on the 17th day. He discontinued treatment after two weeks. Watch 15-40, whispers 4-10.

Before closing I would like to say that the treatment of acute inflammation of the middle ear, arising as a sequel in any of the exauthemata, or from any cause whatever, more particularly in those cases where a collection of fluids (pus or serum) in the middle ear can be made out, should be based upon the rules which guide us in evacuating pus, etc., from any of the servus or abscess cavities situated elsewhere in the body. The indications, then, are to incise the membrana tympanum; completely removing the pus, etc., from the middle ear; irrigate with mild antiseptic solutions; maintain the patency of the opening in the drum for through and through drainage, and keep the tympanic cavity perfectly clean—this line of treatment in the vast majority of cases will not only decrease the mortality from cerebral abscesses, but will prevent a chronic discharge from occurring, and also restore the hearing to almost the normal standard.

The inference to be drawn, then, is that the pus, serum, etc., present in the middle ear should be treated like an abscess cavity anywhere else in the body, of course due regard and consideration being observed for the position, character of structures and accessability of the parts.

A purulent or catarrhal accumulation should be evacuated, antiseptically cleansed and drainage established. The inflammation should not be permitted, as is frequently done, "to rise" and rupture the drum, prolonging the pain and suffering of the patient. The rupture of the drum is uncertain, rarely affording complete drainage, thereby permitting the discharge to collect and increasing the danger of invalvement by continuity of the mastoid cells and antrum. The collection by pressure may produce some permanent damage to the internal ear structures to such an extent that the loss of hearing is complete and irremediable.

The indications, then, to bear in mind in treating this class of patients, are to establish free drainage, which will prevent a chronic otorrhea and minimize the dangers of mastoid complications and keep the middle ear cavity aseptically clean, contributing in materially aiding in the preservation of hearing.

As soon as the discharge ceases and the inflammation subsides, and the incision in the membrana tympanum heals, passive motion of the ear-ossicles and drum should be made in order to prevent anchylosis of the former and retraction of the latter from taking place.

602 Main Street, Houston, Texas.

